# Detection and characterization of the *Pichia manshurica* biofilm on the traditionally produced homemade apple vinegar

**DOI:** 10.55730/1300-0527.3640

**Published:** 2023-10-18

**Authors:** Zeynep İŞLEK KÖKLÜ, Pınar AKKUŞ SÜT, Esra ESKİHORAN ÜÇÜNCÜOĞLU, Sadık KALAYCI, Fikrettin ŞAHİN

**Affiliations:** Department of Genetics and Bioengineering, Faculty of Engineering, Yeditepe University, İstanbul, Turkiye

**Keywords:** Biofilm, bacterial cellulose, *Gluconobacter oxydans*, *Pichia manshurica*, apple vinegar, homemade

## Abstract

*Pichia* yeasts are capable of forming biofilms during vinegar production and causing spoilage in various beverages. In addition, there exists a significant likelihood of encountering yeast contamination which can prevent vinegar production. The present study investigates the detection and characterization of the *Pichia manshurica* (*P. manshurica*) biofilm on traditionally produced homemade apple vinegar. The unique characteristics of vinegar were analyzed with a focus on the constituent, known as the “mother of vinegar”, whose composition is comprised of cellulosic biofilm and acetic acid bacteria, including *Gluconobacter oxydans* (*G. oxydans*) Briefly, *P. manshurica* was isolated from apple vinegar and characterized in terms of the effect of biofilm formation on the surface of the cellulosic film on vinegar production. Microbial identification of vinegar with/without contamination by *P. manshurica* was analyzed through MALDI-TOF mass spectrometry (MS), and biofilm was characterized by Fourier transform infrared spectroscopy (FT-IR), Scanning electron microscopy (SEM), and crystal violet staining. Accordingly, MS spectrum of isolates was identified as *G. oxydans* and *P. manshurica* with a ratio of 2.01 and 1.94, respectively. The FTIR analysis indicated that the peaks within the range of 1150–900 cm^−1^ revealed a high content of polysaccharide in *P. manchuria-*contaminated biofilm, which is attributed to the stretching vibration of C-C and C-O bonds. The spectral region from 2921.51 to 2853.71 cm^−1^ exhibited the characteristic of lipids in bacterial cell walls and membranes. SEM images of bacterial biofilms revealed a three-dimensional network composed of ultrafine fibers with a ribbon-like shape; however, the condensed reticulated structure was observed in contaminated biofilms. The presence of two microbial populations was detected regarding the morphological analysis. Crystal violet staining of contaminated-cellulosic biofilms visualized bacterial and yeast colonization. Concisely, this study emphasizes that the proliferation of *Pichia* during apple fermentation has the potential to adversely affect the quality of the homemade vinegar, due to its distinct biofilm characteristics.

## 1. Introduction

Vinegar is a popular condiment that is widely used in cooking, food preservation, and as a natural cleaning agent. It can be produced from a wide variety of fermentable carbohydrate sources, such as fruits, honey, grains, and starchy vegetables. It is commonly used as a food preservative, and according to the Food and Drug Administration (FDA), it contains 4% acetic acid that is produced through the fermentation of sugary or alcoholic materials [1,2]. Vinegar comprises diverse quantities of stable fruit acids, salts, pigments, and volatile components, such as esters and phenolics, which give it its characteristic aroma and flavor [3]. Apple cider vinegar is widely known for its therapeutic properties and health advantages. It is rich in trace minerals and vitamins, such as vitamins A, C, E, and B, beta carotene, and bioflavonoids, which play a crucial role in cellular function [4]. Vinegar possesses various properties, including antiglycemic, antihypertensive, antimicrobial, antioxidant, and antitumor activities. Additionally, vinegar serves as a fundamental component in Ayurvedic preparations [4].

In Türkiye, there are various types of local vinegar that are specific to different regions. Apple vinegar, which has a history dating back more than 3500 years, is a typical and characteristic product in Türkiye. Traditionally, vinegar has been produced through a slow, natural, and spontaneous fermentation process involving acetic acid bacteria [5]. Besides, fruit juices have conventionally been used as a source for vinegar production in both household and industrial settings, in a two-stage procedure. Initially, fruit sugars undergo fermentation by yeasts, predominantly *Saccharomyces ellipsoideus* (*S. ellipsoideus*), and *S. cerevisiae*, resulting in the production of alcohol. Subsequently, acetic acid bacteria facilitate the oxidation of ethanol and any remaining or supplemented sugars into acetic acid [4,6]. These gram-negative or gram-variable organisms are ellipsoidal to rod-shaped, 0.6–0.8 μm × 1.0–0.4 μm in size [4]. It is possible to find pleomorphic forms that are spherical, elongated, distended, curved, or filamentous. They can convert substrates like glucose, ethanol, lactate, and glycerol into acetic acid. The classification of bacteria into two primary genera, namely *Acetobacter* and *Gluconobacter*, is based on their capacity to overoxidize acetate or lactate, as well as the arrangement of their flagella. Additionally, these are known to produce a film or pellicle composed of cellulose. The acetic acid bacteria and yeasts in the fermentation system become intertwined with the cellulosic pellicle to create the “mother of vinegar” [4]. *Pichia manshurica* (*P. manshurica*) is a yeast species that has been reported in various fermented foods, such as table olives, cocoa beans, wine, and vinegar. The documented spoilage of wine is attributed to the release of volatile phenols and cadaverine. *P. manshurica* exhibits a high degree of adaptation to the wine environment, although it is vulnerable to cleaning agents. Aerobic yeast species, including *Pichia*, which are deemed undesirable, have the capability to develop a film layer at the interface of air and wine upon exposure to air. This microorganism is capable of colonizing filter surfaces, thereby leading to contamination in wineries, as has been documented in previous studies [7,8]. *P. manshurica* poses a significant challenge due to its resistance to organic acids, resulting in the production of undesirable sensory attributes such as odors, tastes, and products. During the process of vinegar production utilizing spontaneous fermentation, there exists a heightened possibility of contamination with the development of biofilm and malodor on the surface of the vinegar. The yeasts that participate in the alcohol fermentation stage may lead to complications by catalyzing the oxidation of alcohol to carbon dioxide and water, as has been previously noted [7]. Therefore, the utilization of yeast strains such as *P. manshurica* in the process of vinegar manufacturing may give rise to complications and is not recommended.

Vinegar production, on the other hand, involves spontaneous fermentation and traditional techniques that are influenced by factors such as water, soil, and climate. These factors determine the quality of the vinegar, and the presence of *P. manshurica* could impact the flavor and quality of the final product [9–12]. In the previous study, yeasts, including *Saccharomyces*, *Pichia*, and *Candida* (*C*.), are categorized into two types based on their function: alcohol-producing and aroma-producing, and are known genera involved in vinegar fermentation. *S. cerevisiae* is the most common alcohol-producing yeast species and dominates the alcoholic fermentation process. Besides, aroma-producing yeasts are less abundant but significantly affect the quality and flavor of alcoholic beverages and vinegar [9]. Therefore, it is important to improve information about the characterization of yeast biofilms such as *P. manshurica*, and to develop methods for their detection in vinegar production to prevent spoilage and ensure the production of high-quality vinegar traditionally produced by cellulose.

In this study, we aimed to explore the microbial detection and characterization of cellulose produced by *Gluconobacter oxydans* (*G. oxydans*), and *P. manshurica*-contaminated cellulosic biofilms in vinegar production, using MALDI-TOF mass spectrometry (MALDI-TOF MS), Fourier transform infrared spectroscopy (FT-IR) analysis, scanning electron microscopy (SEM), and crystal violet staining. Therefore, besides highlighting the detection of *P. manshurica* contamination in the cellulosic biofilms, it sheds light on the comparative characterization of bacterial cellulose used in vinegar production with the biofilm formed as a result of contamination.

## 2. Materials and methods

### 2.1. Materials

The sweet apples were collected from Edremit in Türkiye. Tryptic soy agar and malt extract agar were purchased from Sigma-Aldrich, Germany. HCCA Matrix was obtained from Bruker, United States. Crystal violet stain was purchased from Fluka, US.

### 2.2. Production of apple vinegar

To prepare the vinegar, 1.75 kg of sweet apples were separated from the stems and kept with 2.5% honey, chickpeas, and drinking water in a 10-L glass jar. The mixture was stirred for 2 weeks using a wooden spoon until the apples sank to the bottom. The jar was then covered with cheesecloth and stored at 25 °C and in a dark environment for 3 to 6 months to be fermented. Over time, it was observed that the water remained on top, while the apples sank to the bottom. As fermentation progressed, a membrane-like cellulose biofilm began to form on the apples, gradually thickening. Once fermentation was complete, the mixture underwent filtration, resulting in the separation of the pulp and vinegar constituents.

### 2.3. Collection of the vinegar samples

**Figure 1:**
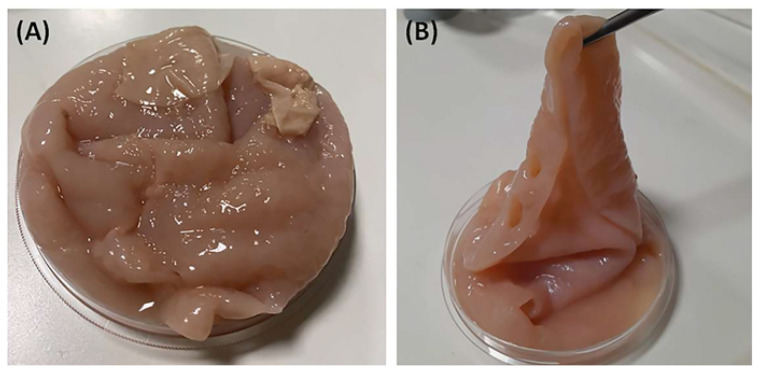
The images of cellulosic biofilm in the apple vinegar.

Homemade apple vinegar was traditionally produced without any pasteurization process or additives (Figures 1A and 1B). Each sample was collected in bottles and stored in laboratory conditions at 4 °C for 24 h.

### 2.4. Microbial identification of the apple vinegar

#### 2.4.1. Isolation of microorganisms from the apple vinegar

The microbiological properties of the vinegar were determined using MALDI-TOF MS. Initially, the samples of the apple vinegar were enriched with alkaline peptone water. Subsequently, serial dilutions to 10^−1^ and 10^−6^ were prepared, and the diluted samples were plated on Tryptic Soy Agar and Malt Extract Agar. These plates were then incubated at 30 °C for 48, and 72 h. Suspicious microorganism colonies were selected from each agar and ultimately purified by reseeding onto fresh Tryptic Soy Agar and Malt Extract Agar plates, followed by additional incubation at 30 °C for 48 and 72 h. The purified colonies were then identified using MALDI-TOF MS (Bruker Daltonik GmbH, Bremen, Germany).

#### 2.4.2. Identification of microorganisms within the apple vinegar using MALDI-TOF MS

Following 48 and 72 h of incubation, the Petri dishes were examined and a single colony structure was identified by matrix-assisted laser desorption/ionization time-of-flight mass spectrometry (MALDI-TOF MS) (Bruker Microflex LT, Bruker Daltonik GmbH, Bremen, Germany). Briefly, a single colony from each Petri dish was transferred onto a MALDI-TOF MS target plate, covered with 1 μL of α-cyano-4 hydroxycinnamic acid (i.e. matrix solution) dissolved with 50% acetonitrile-2.5% trifluoroacetic acid (HCCA matrix), and left to dry at room temperature before protein analysis by MS. Subsequently, it was covered with the target, and analyzed by the mass spectra represented in term of the mass change ratios (m/z) of each microorganism. According to the library matching, the MS spectrum of unknown microbial isolates was compared with the MS spectra of known microbial isolates contained in the MALDI-TOF MS database. In order to detect the species level identification of microbes, a typical mass range m/z of 2–20 kDa was used by Flex Control software, in which mainly ribosomal proteins were represented together with housekeeping proteins.

### 2.5. Characterization studies of the biofilm

#### 2.5.1. FT-IR analysis

Dried biofilm samples were analyzed by FT-IR spectrometer (Thermo Scientific, NICOLET iS50 FT-IR Spectrometer, USA) within the spectral region of 4000–400 cm^−1^. Data collection and processing were performed with OMNIC software, which was provided along with the instrument.

#### 2.5.2. Morphological analysis by SEM

SEM (Zeiss EVO40, Germany, USA) was used to visualize the surface structure of the biofilm samples at an accelerated voltage of 10 kV. Briefly, the dried samples were placed onto a metallic stub and subjected to gold palladium sputtering (EM ACE200, Leica) for 45 s. The prepared samples were then examined under SEM (Zeiss EVO40). The experiments were repeated with three independent studies.

### 2.6. Crystal violet staining

Crystal violet staining solution (0.5%) was prepared in accordance with the previously described procedure [13]. Briefly, 0.5 grams of crystal violet powder was dissolved in 20 mL of methanol and the ultimate volume was adjusted to 100 mL with distilled water. Afterwards, small pieces were cut from the biofilm structure with the help of a scalpel. Biofilm pieces were treated with a crystal violet dye solution for 10 min. After staining, the biofilm pieces were washed 3 times for 5 min to remove excess dye. The stained biofilm pieces were taken on a glass slide and visualized with bright field imaging using an inverted microscope (ZEISS Axio Vert.A1).

## 3. Results

### 3.1. Microbial identification using MALDI-TOF MS

In accordance with the library database of MALDI-TOF MS, the MS spectrum of unknown microbial isolates was identified as *G. oxydans* ssp*. B544 UFL*, *G. oxydans* ssp*. B540 UFL*, and *P. manshurica* with a ratio of 2.01, 1.94, and 1.70, respectively.

### 3.2. FT-IR analysis of the cellulosic biofilm samples

**Figure 2:**
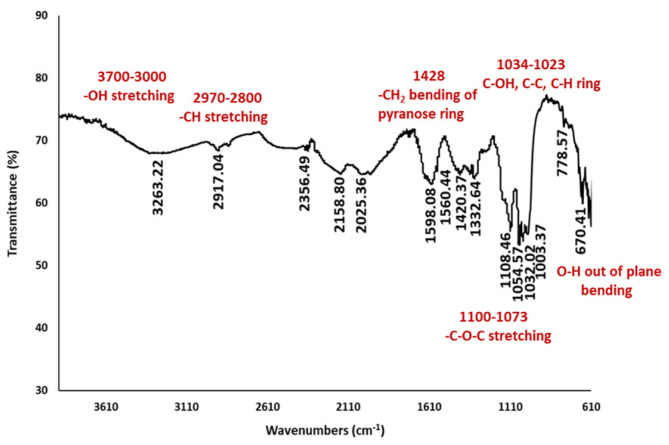
FT-IR spectra of cellulosic biofilm.

Cellulosic biofilm produced by *G. oxydans* was analyzed by FT-IR to identify the chemical structure, as shown in Figure 2. Accordingly, cellulose-specific characteristic peaks in the range of 4000–400 cm^−1^ were observed in the FTIR spectrum of bacterial cellulose. The cellulosic sample indicated the characteristic peaks at 3263 cm^−1^ for O-H stretching vibration, at 2917 cm^−1^ for C-H stretching vibration, at 1420 cm^−1^ for C-H bending vibration, at 1032 to 1054 cm^−1^ for C-O-C and C-O-H stretching vibration of the sugar, and at 670 cm^−1^ for the OH out-of-phase bending vibrations (Figure 2).

### 3.3. FT-IR analysis of the *P. manshurica*-contaminated cellulosic biofilm

**Figure 3:**
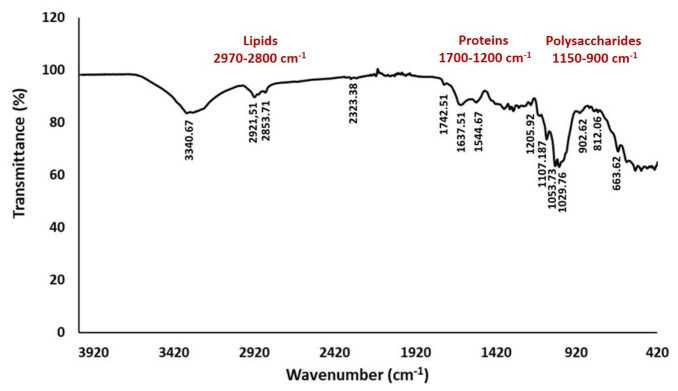
FT-IR spectra of *P. manshurica*-contaminated cellulosic biofilm structure.

The *P. manshurica*-contaminated cellulosic biofilm structure was examined by FT-IR spectroscopy within the spectral region range of 4000–400 cm^−1^, in which the presence of chemical groups belonging to important components such as proteins, lipids, and polysaccharides was investigated (Figure 3). The peaks in the range of 1150–900 cm^−1^ indicated high polysaccharide content, in which the band region was mainly formed as a result of stretching vibration of C–C and C–O bonds and deformation of C–O–H and C–O–C bonds (Figure 3). The stretching vibration of the lipids was observed between 2921.51 and 2853.71 cm^−1^ (Figure 3).

### 3.4. Morphological analysis of the biofilms

**Figure 4:**
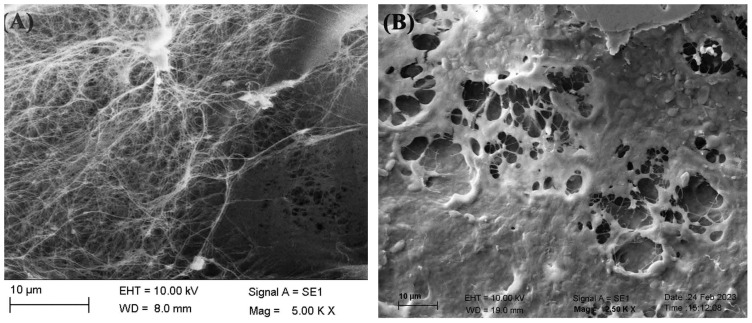
SEM images of **(A)** Bacterial cellulose; and **(B)** *P. manshurica*-contaminated cellulosic biofilm. Scale bars correspond to 10 μm.

**Figure 5:**
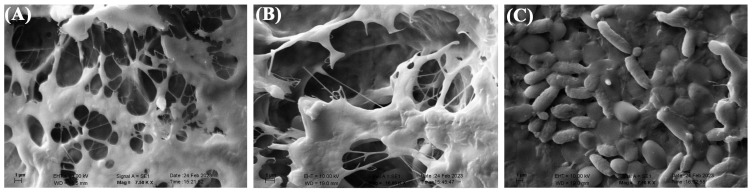
SEM images of **(A&B)** *P. manshurica-*contaminated cellulosic biofilm; and **(C)** *P. manshurica* yeast and *G. oxydans* bacterial populations. Scale bars correspond to 1 μm.

The SEM images of cellulosic biofilm samples indicated a three-dimensional porous network structure consisting of randomly arranged ribbon-shaped ultrafine fibers (Figure 4A). The filaments and reticulated structures of contaminated cellulosic biofilms from *G. oxydans* and *P. manshurica* differ morphologically (Figures 4 and 5). Accordingly, bacterial cellulose was observed to lose its reticulated structure and nanofiber network, and was replaced by the structure of the *P. manshurica* biofilm (Figures 4B, 5A, and 5B). As a result of the convergence by thick layers, nanofibers were unable to be observed, indicating that the topology of the pore network was highly interconnected. Furthermore, two different microbial populations were visualized under the SEM images, in which the spherical or ovoid shape of micrometers in size, occurring singly, in pairs, in chains, or in groups, corresponds to *P. manshurica* yeast populations, whereas *G. oxydans* was detected with ellipsoidal to rod-shaped bacterial properties (Figure 5C).

### 3.5. Crystal violet staining

**Figure 6:**
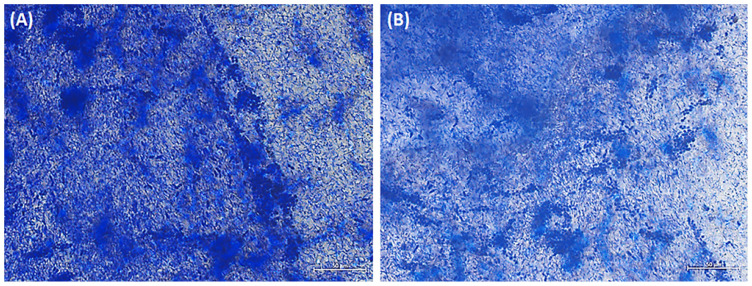
**(A&B)** Brightfield microscopy images of *P. manshurica-*contaminated cellulosic biofilms after crystal violet staining. Scale bars correspond to 50 μm.

Cellulosic biofilms contaminated with *P. manshurica* were observed under brightfield microscopy, revealing the development of a layer of positive cells that exhibited crystal violet staining on the surface of the cellulosic biofilm (Figure 6). In parallel to SEM images, there was a development of filamentous *G. oxydans* bacterial and *P. manshurica* yeast colonization, resulting in the formation of a complex filamentous structure that was abundantly dispersed without showing any selective binding between the different surfaces of the biofilm (Figures 6A and 6B).

## 4. Discussion

Cellulose, which is used extensively in the paper and textile industry, can be obtained from plant sources (containing 60–90% cellulose), as well as produced by microorganisms such as bacteria, fungi, and algae. The high purity, mechanical properties, and high water-holding capacity of bacterially produced cellulose are advantageous for its use in different areas. Since the reporting of *Acetobacter xylinum* as a producer of cellulose, many cellulose-producing bacterial strains have been described previously [14,15], including *Azotobacter*, *Gluconacetobacter*, *Pseudomonas*, and *Rhodobacter* [16]. In the present study, *G. oxydans*, which is known as one of the cellulose-producing microorganisms, was determined by MALDI-TOF MS. Previously, simple sugars in the apple such as glucose, fructose, and sucrose were found be as important substrates that contribute to traditionally produced apple vinegar bacterial cellulose production, owing to its high sugar content, thus enhancing a favorable environment for cellulosic “mother of vinegar” production [15]. Together with the sugar source of the apples, *Gluconobacter* spp. has taken advantage of cellulose production [4]. In the process of vinegar production, there is a high risk of specific yeast contamination, including *Pichia* spp., involved in the alcohol fermentation, resulting from the oxidation of alcohol to CO_2_ and H_2_O [7]. *Pichia* species can lead to biofilm formation and excessive foul odor on the surface of vinegar production [8]. For this reason, it is necessary to determine the microorganism in the contamination, characterize the biofilm formation from *Pichia*, and perform its instrumental and morphological analysis, including MALDI-TOF MS, FT-IR, SEM, and crystal violet staining.

### 4.1. The chemical structural analysis of cellulosic biofilms by FT-IR

In the present study, the examination of microbial biofilm structures by FT-IR spectroscopy was carried out in the spectral region range of 4000–400 cm^−1^, which indicates the presence of chemical groups belonging to important components such as proteins, lipids, and polysaccharides. Previously, FT-IR biofilm analysis demonstrated that lipids, proteins, and polysaccharides were absorbed in the specific spectral ranges, including 3000–2800 cm^−1^, 1705–1600 cm^−1^, and 1200–950 cm^−1^, respectively [17,18]. Furthermore, in another study, the peaks of protein content were detected as amide I at 1600–1700 cm^−1^, amide II at 1500–1600 cm^−1^, and amide III at 1200–1350 cm^−1^ [17]. In addition, it has been reported that protein units exhibit IR band properties through C=O stretching at the amide I region, N–H bending and C–N stretching at the amide II region, and C–N bending and N–H stretching at the amide III region [17,19,20]. On the other hand, the peaks in the range of 1150–900 cm^−1^ indicated high polysaccharide content, in which the band region is mainly formed as a result of stretching vibration of C–C and C–O bonds and deformation of C–O–H and C–O–C bonds [21,22]. The peaks of symmetrical and asymmetrical C-H vibrations were observed between 2800 and 2970 cm^−1^, indicating the presence of lipid molecules in EPS due to the characteristic of lipids condensed in bacterial cell walls and membranes [23,24]. Similarly, in our biofilm sample, the stretching vibration of the lipids was observed between 2921.51 and 2853.71 cm^−1^ (Figure 3).

### 4.2. The morphological analysis of cellulosic biofilms by SEM

The SEM images of cellulosic biofilm samples were visualized as three-dimensional porous network structures and randomly arranged ribbon-shaped ultrafine fibers (Figure 4). Saxena et al. reported that cellulose synthesis was characterized by crystallization unidirectional growth, in which glucose molecules were bounded β (1–4) glucosidic bonds linearly [25,26]. Oriented microfibrils were formed by the union of glucosidic chains via intramolecular hydrogen bonds. The diameter of thin filaments was arranged between 34.34 and 39.16 nm, which is smaller than plant cellulosic fibrils [26,27]. The results were consistent with the previously reported cellulose structure [9,26,28]. The filaments and reticulated structures of contaminated cellulosic biofilms from *G. oxydans* and *P. manshurica* display differences morphologically (Figures 4 and 5), wherein the *P. manshurica-*contaminated cellulosic biofilm lost its reticulated structure, and nanofiber network, resulted in the formation of thick layers rather than nanofibers. Kim et al. (2006) stated that the vast surface area of cellulose was essential for the attachment of cellulose and the development of vascularization [29]. In addition, within the range of 10–100 nm, cotton fibers, wood slurry fiber, and synthetic fiber were indicated by Youshinaga et al. [30], which could be linked to the unique and fine structure of the cellulosic biofilms from *G. oxydans*.

### 4.3. The process of staining biofilm-populated biomaterial using crystal violet

The staining technique for the microscopic examination of biofilms offers the benefit of efficiently tracking the development of biofilms on physical substrates. The present investigation employs a combination of qualitative and semiquantitative methodologies to identify biofilms through an advanced stereomicroscope, which enables a comprehensive analysis of the structure and function of biofilms. In addition, the utilization of mosaic technology has the potential to be implemented for the examination of extensive surfaces extending square centimeters, in contrast to conventional methods which focus on considerably smaller regions.

Basically, crystal violet is a cationic dye commonly utilized in microbiology to label negatively charged surface molecules such as peptidoglycan and extracellular polysaccharides [31]. To mitigate the potential for manipulation and artifacts resulting from the bacteria and their biofilm, a direct application of crystal violet staining was employed on the biofilm surfaces. The prominent technique to evaluate biofilm formation generally involves the use of a 96-well microtiter plate assay [32], wherein the detection of the dye taken from the formerly stained biofilm was typically accomplished through colorimetric means. Although crystal violet staining is generally applied in a microtiter plate assay [33,34], it was carried out in this study by directly staining the biofilm sample taken from the vinegar-producing environment and examined using brightfield microscopy (Figure 6).

A previous study demonstrated that bacterial biofilm structures consist of an extracellular matrix containing long-chain polymeric molecules, mainly negatively charged exopolysaccharides and lipopolysaccharides, and secreted by related cells [35]. Negatively charged surface molecules in the EPS structure and cells were visualized using crystal violet staining to identify biofilm-producing organisms and further visualize the biofilm formations. Accordingly, the images indicated that the filaments were stacked on top of each other and formed a structure in which bacterial and yeast cells were observed locally, which could be distinguished considering their morphologies, including rod-shaped (i.e. *P. manshurica* yeast) and spherical-shaped (i.e. *G. oxydans* bacteria) (Figures 6A and 6B). In addition, *P. manshurica* biofilm formation was achieved to be observed from a vinegar-producing environment stained with crystal violet under the bright field microscope, according to the dense structure of biofilm and hence the dye density.

Given that SEM images and crystal violet staining are not able to identify bacterium strains as part of a biofilm, our technique was combined with MALDI-TOF MS and FT-IR analysis. However, the limitations of crystal violet staining in identifying bacterial strains within biofilms necessitate the use of molecular assays to achieve more accurate identification of the strains involved in our approach. Therefore, further investigation is required to establish a precise relationship between colony-forming unit counts and/or polymerase chain reaction analysis. This will enable the conversion of our qualitative read-out into more quantitative data.

## 5. Conclusion

The presence of *Pichia* species has been observed to result in the development of biofilm and malodorous compounds on the surface of the bacterial cellulosic film during production. As a result, yeast contamination may develop in the process of vinegar manufacturing and may give rise to complications. Based on the experimental results of the detection and characterization studies, including MALDI-TOF, FT-IR, SEM, and crystal violet staining, this study highlights the potential detrimental effect of *Pichia* proliferation on the quality of homemade vinegar during apple fermentation, due to its unique biofilm characteristics. Consequently, this study emphasizes that the proliferation of *Pichia* during apple fermentation can potentially have a negative impact on the quality of the homemade vinegar, owing to its distinct biofilm characteristics, and therefore necessitates identifying the detection and characteristic features belonging to the microbial source.
